# Improving Care Transitions: An Initiative between the Emergency Department and Senior Care Facilities

**DOI:** 10.51894/001c.26862

**Published:** 2021-08-30

**Authors:** Michelle “Joan” Moccia, Daniel Keyes

**Affiliations:** 1 Emergency Medicine St Mary Mercy Hospital, Livonia, MI

**Keywords:** health information, senior care facilities, emergency department, transitions of care

## Abstract

**INTRODUCTION:**

The transfer of individuals (i.e., residents) between senior care facilities (SCF) and the emergency department (ED) remains an ongoing healthcare quality gap as communication of key resident information is often lost. For this study, a sample of SCF representatives were invited to join a collaborative group termed Safe Transition of All Residents For yoU and Me (STARForUM, STAR-F) to improve SCF resident transitions of care.

**STUDY PURPOSE:**

The purpose of this pilot study was to invite a convenience sample of SCF facilities to join a collaborative intervention named *Safe Transition of All Residents For yoU and Me* (STARForUM, STAR-F) to improve information exchange during SCF residents’ transitions of care. The potential influence of a hospital-SCF collaboration program to improve transfer of essential SCF resident information sent to the hospital ED was used as an evaluation measure.

**METHODS:**

This study project enrolled a total of 120 residents (i.e., patients) with 40 (33%) transferred from participating STAR-F facilities.

**RESULTS:**

Following the authors’ development of a transfer checklist, STAR-F facilities sent a significantly greater number of essential elements comprised of the resident’s medical history information to the ED compared to non-STAR-F facilities. Controlling for the standard classification of skill level of the individual facility, STAR-F residents had significantly higher essential information transmission composite scores (10.5 + 2.9 for STAR-F patients vs. 7.75 + 3.1 for non-STAR-Fs p = < 0.01) that may have served to reduce number of associated transition errors.

**CONCLUSIONS:**

The findings of this study suggest that a collaborative hospital-SCF initiative can significantly improve transfer of information for elderly residents during ED visits, help guide clinical decision-making and optimize care coordination.

## INTRODUCTION

Numerous studies have shown that mutual exchange of key medical care information is frequently substandard between emergency departments (EDs) and long-term care settings.^1,2^ Healthcare facilities and EDs traditionally operate independently due to their different organizational arrangements and priorities. The sharing of transfer information between ED providers from senior care facilities (SCFs) (e.g., skilled nursing (SNF), assisted living facilities (ALF), and independent living facilities (ILF)) remains frequently inconsistent or incomplete, predisposing residents to negative health outcomes.^2^

Earlier studies have indicated that up to 10% of nursing home residents are transported to EDs without any pertinent documentation, and essential information (e.g., reason for transfer, vital signs, code status, medication lists, or baseline mental status) is often not provided for the remaining 90% of ED visits.^3,4^ The absence of key information such as medical and surgical history, advance directives, baseline mental and functional status, or medications and allergy history can result in unintentional medical errors such as unwanted resuscitation, adverse drug reactions or unrecognized delirium.^3^

Moreover, it logically follows that inadequate information forces a broader differential diagnosis, oftentimes unnecessarily increasing the number of costly tests, procedures, and length of stay.^5^ As our nation’s population ages, the proportion of the aged residing in SCFs will increase.^6^ According to the National Center for Assisted Living (NCAL), there are over 36,000 licensed assisted living facilities (ALF) nationwide with an estimated one million residents making their home in ALF/residential care communities, including about 131,000 receiving assistance under the Medicaid program.^6^ In 2012, approximately 5% of the elderly population reside in a nursing home, although a much larger proportion, 25-40%, will also require institutionalization at some point in their lives.^7^ In 2004 alone, there were 2.7 million ED visits by residents of nursing homes or other institutions.^8^

Unnecessary transfers of care remain an area of potential harm for such elderly.^2,9,10^ Inadequate communication increases utilization not only in the ED, but also with hospital inpatient, post-acute care and ambulatory services.^11–13^ Unknown End-of-life (EOL) care decisions can cause confusion and negatively affect care among ED providers and other hospital personnel.^14^ Potential unwanted or unnecessary EOL care may occur when immediate resuscitation is required, and a medical order (e.g., code form or AD information) is absent. According to Terrell et al. in 2009, incomplete data contribute to a flawed care plan, medication errors, and re-hospitalization thus further increasing health care costs.^15,16^

A high rate of recidivism (i.e., ” bounce back”) in older adults after ED visits have been reported, with insufficient care plans and discharge processes identified as important influences of more frequent ED transfers for the elderly.^17^ In one 2016 study, 170 (23%) of a cohort of 739 Medicare post-acute care patients were readmitted to the hospital within 30 days of being transferred to a skilled facility.^18^

Additionally, patients with delirium during their hospital stay have an increased odds of readmission to the hospital.^19^ Delirium may be present in up to 40% of patients transferred to the ED from long term care facilities and was associated with several poor health outcomes including malnutrition, fluid and electrolyte disturbances, aspiration pneumonia and pressure ulcers.^20^ Due to normal aging processes, older adults are also frequently predisposed to adverse drug events (ADEs). An accurate medication history or record are therefore also essential to avoid incomplete medication reconciliation and potentially fatal hospital and post-discharge ADE.^21–24^

In summary, the US healthcare system has developed independent, semi-autonomous “silos” that do not communicate adequately to ensure safe transitions of care.^13,14,25^ The Society for Academic Emergency Medicine Geriatric Task Force recognized quality gaps in transitions of care in both directions between facilities and the ED and developed quality indicators for transitional care.^10^ Before this study, the authors concluded that such improvement strategies could ideally be used to improve fragmented transfer care that was apparent following the opening of their Senior ER in Michigan.

### Purpose of Study

This pilot project was designed to initially assess the feasibility and efficacy of a SCF-to-ED transfer checklist to facilitate transfer of key documentation and information. For the purposes of this study the terms “senior,” “elderly” and “older” adults were those considered to be 65 years of age and older. Specifically, it was hypothesized that facilities that participated in the STAR-F program by using the transfer checklist would improve the number of key elements sent to the hospital ED compared to those facilities that did not adopt the checklist.

## METHODS

### Design, setting and sample

The study hospital had a total of 304 beds and 53 ED beds with an annual ED visit volume of approximately 45,000 patients. Patients who were 65 and older represented 33% of the hospital population area with 22% of this population residing in one of the 33 SCFs surrounding the hospital. Before data collection, the study design had been reviewed and approved by the authors’ hospital IRB as “non-human subject” research.

Those residents included were 65 years age or older, resided in a SCF in the hospital catchment area and were transported specifically to the study site. If the resident situation was of such high acuity to not permit sufficient time to complete the transfer form upon EMS arrival, the information was to be faxed to the ED once EMS departed the facility.

### Project Recruitment

To help improve communication between SCFs and the ED at a local level, an invitation was sent to the surrounding 33 SCFs. A group Safe Transition of All Residents For yoU and Me (STARForUM, STAR-F) emerged to collaborate on initiatives to improve information transfer. Similar standardized transfer documents for communication of key information had been shown to be effective in the quality improvement program interventions to reduce care transfers (INTERACT).^7,8,12^ However, the authors were unable to identify any earlier transition of care studies that simultaneously involved different care settings (ILF, ALF and skilled).

A convenience sample of 45 stakeholders responded to an emailed invitation to find out more about the study program. During one of the informational meetings, the authors decided on the STAR-F name. This hospital created a community transition collaborative group that met together monthly to improve bi-directional transition of care. The collaborative group consisted of an interdisciplinary care team (ICT) that met together monthly to improve bi-directional transition of care between EMS, EDs and SCFs. (Table 1)

**Table 1.** Description of STAR-F Meeting Participants and Facilities

Independent Living Facility (ILF)	Hospice Care
Assisted-Living Facility	Social Worker
Skilled Nursing Facility	Case Manager
Licensed Home Care	ED Registered Nurse
Non-licensed Home Care	Local EMS
Palliative Care	Nurse Practitioner

This one-page STAR-F transfer checklist form that was developed using SBAR (i.e., Situation, Background, Assessment, and Recommendation) approach emerged from identified ED essential elements to facilitate decision, diagnosis and disposition. (Appendix A) To help identify transition gaps, a series of process flow diagrams were developed to depict each facility’s capabilities and define key transition information exchange steps. (Figures 1 through 3 in Appendices B, C, and D)

### Procedures and Measures

There were two phases to this study. Initially, SCF participants were asked to create a transition checklist of mutually agreed upon essential elements followed by use of the transfer checklist sent with their resident to the ED. The project sample included all residents (i.e., patients) transported from a local SCF to the study hospital ED as their 911 provider of care during the three-month study period between November 19, 2013 and February 14, 2014 (n = 120). Facilities were classified as either STAR-F or non-STAR-F based on whether they participated in the monthly forum meetings.

SCF resident baseline measurements included gender, age, facility type and key elements obtained from transfer documents (Table 2). Program compliance was primarily measured using an original 15-point scale of key elements on the STAR-F transfer checklist developed by the authors with input from an expert panel of approximately 35 ED nurses and physicians along with 45 SCF personnel.

**Table 2.** Facility type and key elements received (%).

Type of facility	*n*	%
Assisted Living Facility (ALF)	33	27.5
Independent Living Facility (ILF)	28	23.3
Skilled Nursing	59	49.2

N = number of facilities; % key elements

Use of the STAR-F transfer checklist was not mandatory if a facility already had a transfer form in place, however all STAR-F members agreed to supplement information absent from current transfer process. Each facility had an equal opportunity to participate in using the transfer checklist. Each element represented a key point of information established as critical for patient care. The primary outcome of the study was the proportion key elements present upon ED presentation. Each element was subsequently evaluated independently to determine which factors provided greater discrimination between facilities.

### Data Analyses

Primary information collected from the transfer checklist included medications, advance directives, facility information, family notification, medical and surgical history, baseline mental and functional status, and any dietary concerns. All data were entered in a secure portal on an Excel spreadsheet. Descriptive analyses were performed and summarized.

Project data were analyzed by the authors’ healthcare system analyst using Statistical Analytics software (SAS, version 9.4, SAS Institute Inc., Cary, NC). Basic descriptive statistics and Student T-test were performed. All data are expressed as numbers (percentages), means and standard deviations. Chi-square was used to calculate equality of two proportions. Student’s *t*-test was used for comparing means of continuous variables. Statistical significance was set at a p value of < 0.05.

## RESULTS

During the three-month implementation period, transfer records from 120 residents sent to the ED were reviewed from SCFs (ILF, N = 23; ALF, N = 33, and SNF, N = 59). The cohort included 40 resident (33.3%) from STAR-F facilities, defined as those who participated in monthly forum meetings, and 80 residents (66.67%) from those not participating (non-STAR-F facilities). Baseline characteristics collected included age, gender, and type of facility. Females constituted 69.17% (n = 83), and mean age was 86. Males comprised 30.83% (n = 37), at a mean age of 83.

There were forty (40.7%) STAR-F patient transfer checklists reviewed and eighty (59.3%) of non-STAR-F transfers checklists. STAR-F facilities were much more likely to transfer more key elements of information than non-STAR-F facilities (means 10.5 ± 2.9 vs. 7.75 ± 3.1, p < 0.01). Controlling for SNF residents only (n = 59), STAR-F revealed a mean composite score of 11.3 ± 2.3 key transfer elements vs. 9.1 ± 2.61 received from non-STAR-F facilities (p < 0.01). The mean number of information elements provided was also significantly greater for STAR-F ALF (11.2 + 3.6) vs non-STAR-F ALF sites (6.9 + 2.6) (p < 0.01). The difference of key elements between ILF was not statistically significant.

### Individual Transition Elements

Four of the key information transition elements (i.e., medication time, code status or presence of AD document, baseline mental and functional status) demonstrated significantly greater receipt of information form STAR-F facilities (i.e., p-value < 0.05). Table 3)

**Table 3.** Summary of Key Documented Elements: STAR-F and non-STAR-F SCFs

**Key elements**	**STAR-F (n = 40)**	**non-STAR-F (n = 80)**	***p-value***
Medication List	39 (97.5%)	69 (86.25%)	0.0528
Medication Administration Record	27 (67.5%)	40 (50.0%)	0.0688
Today's Medication Times	23 (57.5%)	26 (32.5%)	**< 0.01**
Allergies	35 (87.5%)	66 (82.5%)	0.4794
Code Status	31 (77.5%)	48 (60.0%)	0.0567
Signed copy Code Status or AD	23 (57.5%)	22 (27.5%)	**< 0.01**
Medical History	33 (82.5%)	62 (77.5%)	0.5249
Surgical History	12 (30.0%)	17 (21.25%)	0.2912
Baseline Mental Status	29 (72.5%)	19 (23.75%)	**< 0.01**
Baseline Functional Status	29 (72.5%)	18 (22.5%)	**< 0.01**
Dietary Concerns	23 (57.5%)	36 (45.0%)	0.1966
Family DPOA Phone number	36 (90.0%)	68 (85.0%)	0.4475
Notification of Family DPOA	16 (40.0%)	20 (25.0%)	0.1553
Facility Phone Number	31 (77.5%)	51 (64.56%)	0.1496
SCF Facility Address	33 (82.5%)	58 (72.5%)	0.2277

AD: Advance Directive<br>DPOA: Durable Power of Attorney<br>SCF: Senior Care Facility

## DISCUSSION

The findings from this pilot study suggest that using the authors’ transfer checklist to improve SCF-to-ED information transfer was feasible. Our results match those of numerous earlier studies demonstrating that similar collaboration including the types of information transferred between hospitals, ED, and other healthcare facilities can prove key for optimal transitions of care for elderly patients.^1,25,26^

Quality improvement interventions such as the INTERACT have earlier demonstrated that tools such as transfer forms and SBAR-formatted structured information forms can improve communication, prevent unnecessary hospitalizations, and reduce related costs and complications.^27,28^ Lack of key information flow causes communication breakdown and predisposes SCF residents to omissions in medical care, unwanted or less care, unnecessary exams, hospitalizations and future readmissions.^11–16^

These current study results demonstrate the utility and feasibility of reaching out to local facilities to work together and create strategies such as the transfer checklist/form to improve gaps in transitions of care. However, there was no statistically significant difference noted with ILFs with the use of the transfer form. This may be because ILF residents are responsible for sharing their own medical information and history with the ED provider and emergency personnel unless they have previously contracted for medical home health services within the facility.

Four key STAR-F information elements (i.e., medication time, code status, baseline mental and functional status) may have assisted in improving transfer of care. The improved provision of medical orders of code status and or presence of AD avoiding wrongful resuscitation may have also informed EOL and ED visit care. Reported baseline mental status and dementia history also aided in delirium detection, a common geriatric syndrome that increases with aging, and is associated with increased lengths of hospital stay and increased mortality.^29^

The accurate description of baseline functional status helped determine fall risk and thus permitted the use of targeted mobility aids in the ED. However, several possible elements of transferred information (e.g., list of medications and the medication administration record) were not significantly different between the intervention and control group. (Table 3).

### Project Limitations

This single-hospital pilot project was not without limitations. The authors did not examine the accuracy of information flow from ED discharge back to SCFs or later resident outcomes. Also, since each SCF had different capabilities and operated under different quality metrics, this may have impacted their capacity to provide the key elements measured during this study. Obtaining key elements such as code status or medication administration record (MAR) from residents in ILF required the resident themselves, not the professional, to provide essential health information.

## CONCLUSIONS

These findings suggest that a structured quality improvement approach to develop a hospital-SCF collaborative can improve SCF-to-ED transfer of critical information in a moderately-sized community-based acute care hospital setting. More controlled stratified research (i.e., SNF vs. ALF vs. ILF) is needed to evaluate the individual impact of these two methods (i.e., cross-facility collaboration and use of a standard transition of care transfer checklist) to improve elders’ transitional care. EMS personnel, often the interim care provider, should also continue to be included in future work to ensure communication of essential information across healthcare sectors prone to fragmentation.

### Conflicts of Interest

The authors have no conflicts of interest to declare.

## Figures and Tables

**Figure:**
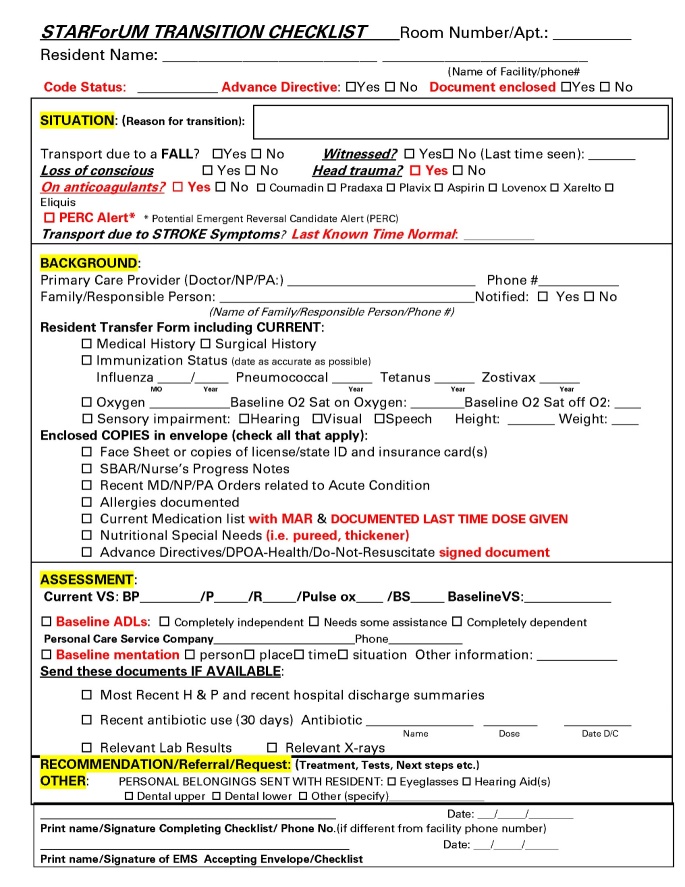
Appendix A

**Figure:**
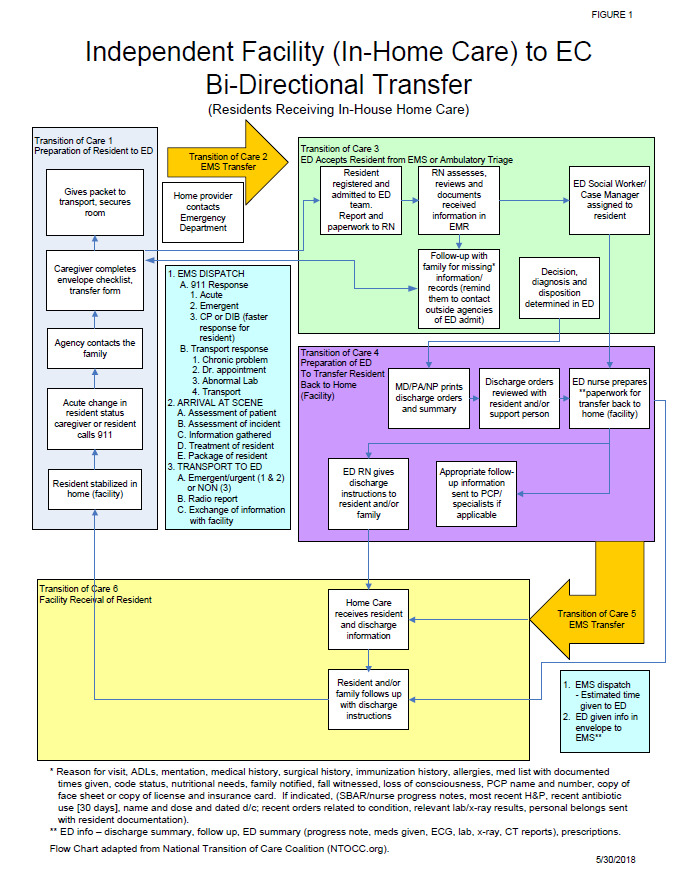
Appendix B

**Figure:**
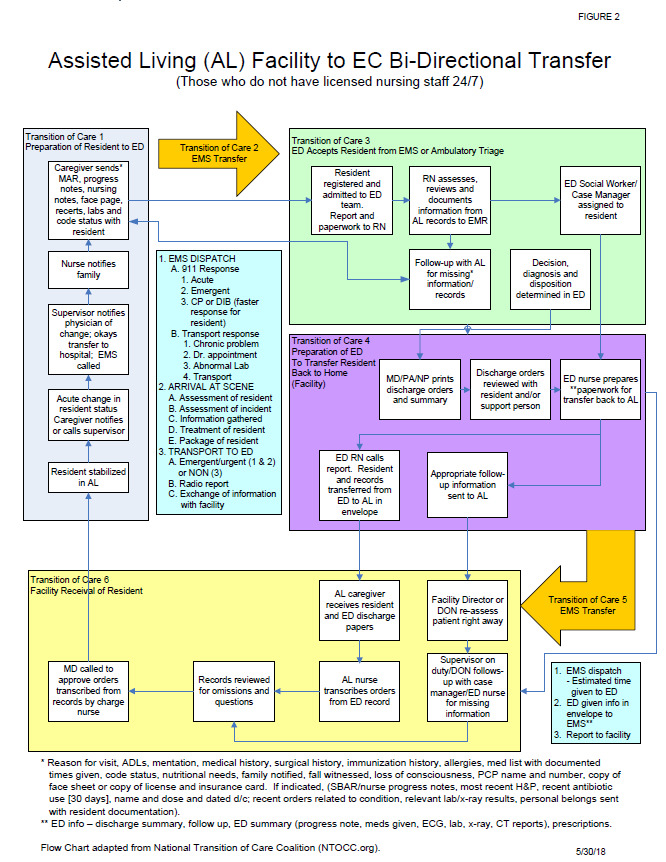
Appendix C

**Figure:**
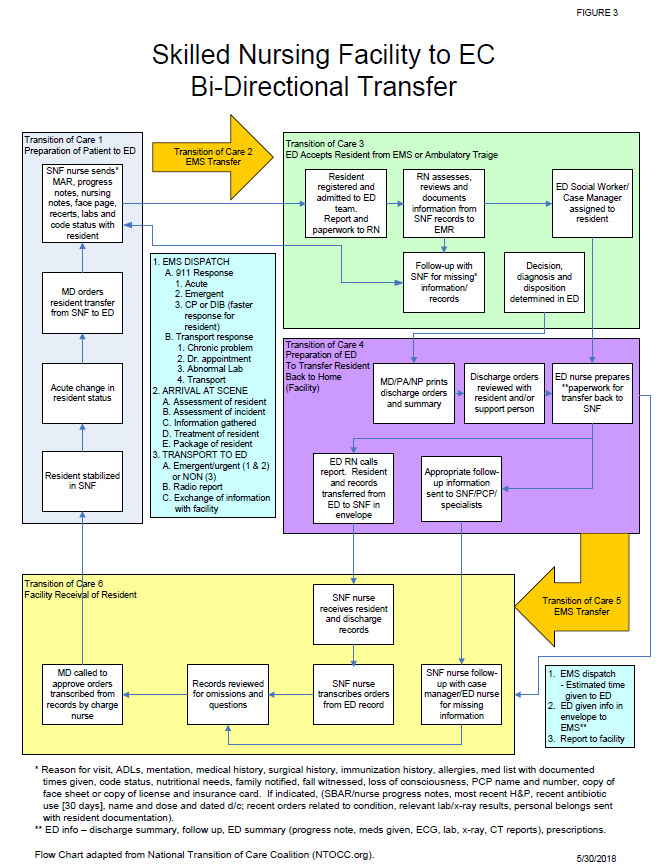
Appendix D
